# Venous malformations involving the buccal fat pad: anatomical extension patterns and management implications

**DOI:** 10.3389/fneur.2026.1931652

**Published:** 2026-09-03

**Authors:** Yanyan Zhang, Jie Liang, Muqing Liu, Meng Liu, Yunan Liu

**Affiliations:** 1 Department of Oral and Maxillofacial Surgery, School and Hospital of Stomatology, Peking University, Beijing, China; 2 Department of Oral and Maxillofacial Radiology, School and Hospital of Stomatology, Peking University, Beijing, China

**Keywords:** buccal fat pad, facial asymmetry, magnetic resonance imaging, sclerotherapy, vascular anomaly, venous malformation, zygomatic arch

## Abstract

**Background:**

Venous malformations (VMs) involving the buccal fat pad (BFP) are uncommon lesions that may cause facial swelling and contour deformity. Although vascular malformations represent the most common pathology affecting the BFP, their anatomical extent, extension patterns, and implications for treatment planning remain poorly defined. This study assessed the imaging features and anatomical extension patterns of BFP VMs and summarized corresponding staged treatment strategies.

**Methods:**

Consecutive patients with VMs involving the BFP treated at a tertiary referral center between 2015 and 2025 were retrospectively reviewed. Clinical presentation, imaging findings, anatomical extent, treatment strategies, and outcomes were recorded. Lesions were classified according to imaging-defined anatomical extension. A narrative literature review was also performed, and previously reported cases were retrospectively reinterpreted with attention to anatomical extent, deep-space involvement, and skeletal asymmetry.

**Results:**

Twenty-two patients were included. Facial swelling or asymmetry was the most common presenting manifestation (21 of 22 patients, 95.5%). Fifteen lesions (68.2%) extended beyond the buccal component, most commonly into the infratemporal and pterygomandibular regions. According to the extension-based classification, seven lesions were categorized as Type I, 10 as Type II, and five as Type III. Image-guided sclerotherapy was performed as the initial treatment in all patients. Additional soft-tissue contouring was performed in 10 patients, and osteoplasty was performed in three. Lesion-related symptoms resolved completely in 17 patients (77.3%) and improved in five (22.7%). Facial symmetry was restored in 15 patients (68.2%) and improved in seven (31.8%). Retrospective reinterpretation of 12 previously reported cases showed similar anatomical extension patterns.

**Conclusion:**

BFP VMs show reproducible anatomical extension patterns, most commonly with spread beyond the buccal component into adjacent deep facial spaces. Recognition of these patterns may facilitate imaging assessment, treatment planning, and staged correction of facial asymmetry.

## Introduction

Venous malformations (VMs) are among the most common vascular malformations, with approximately 40% occurring in the head and neck region. Sclerotherapy has gradually become a mainstay of treatment because it is minimally invasive and allows preservation of adjacent functional structures. However, treatment planning remains challenging when deep or infiltrative lesions extend into complex anatomic spaces and are associated with facial contour deformity (1, 2).

**Figure 1:**
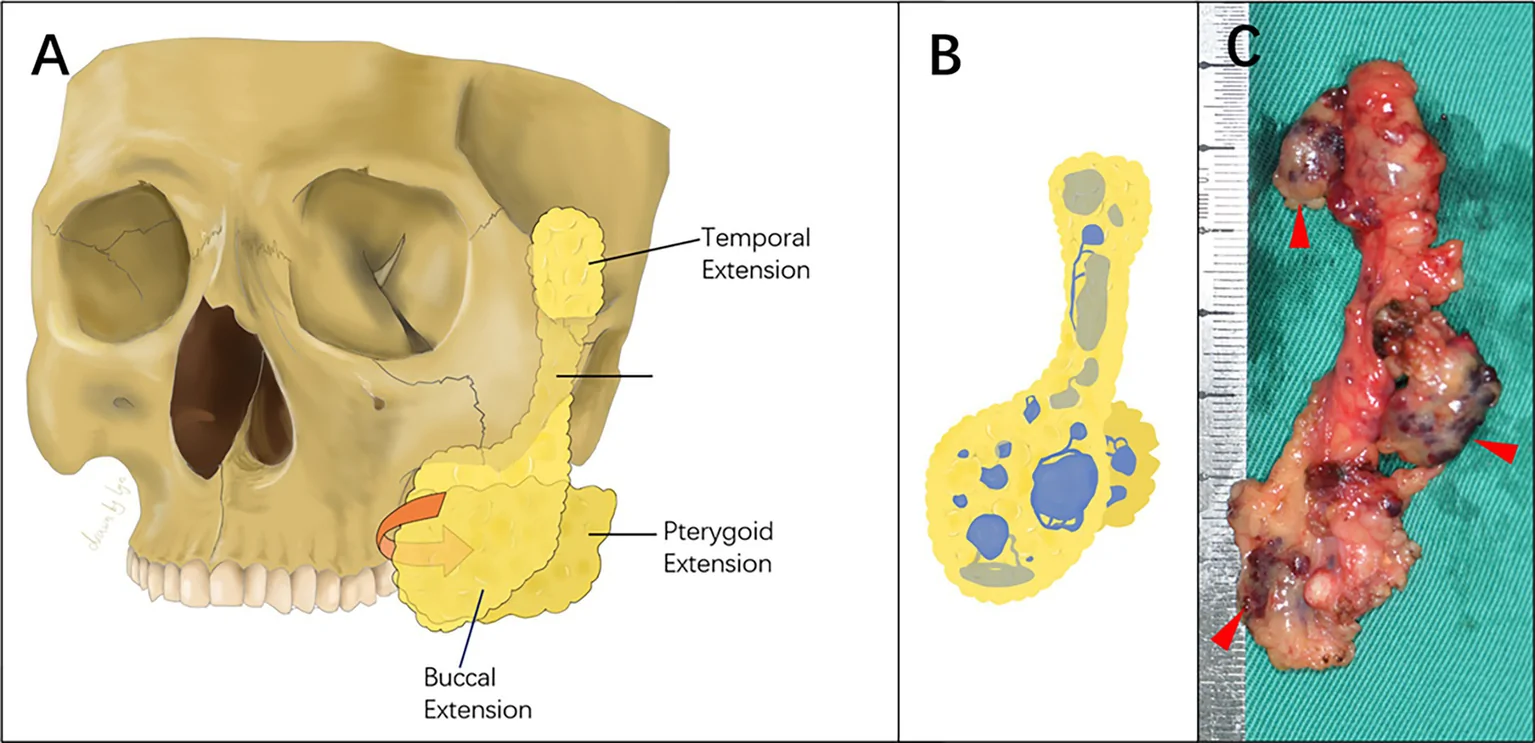
Anatomy and compartmentalized involvement of the buccal fat pad by venous malformation. **(A)** The buccal fat pad consists of a central body and major extensions, including the buccal, temporal, and pterygoid extensions. **(B)** Multiple venous spaces are distributed within separated fat lobules and extensions of the buccal fat pad. **(C)** Gross specimen shows separated venous malformation components within intervening fibrofatty tissue. Arrowheads indicate discrete vascular nodules.

The buccal fat pad (BFP) is a distinct deep facial structure rather than a simple adipose tissue compartment (3, 4). It consists of a central body and several extensions, including the buccal, pterygoid, and temporal extensions, which communicate with adjacent deep facial spaces such as the infratemporal and pterygomandibular regions (Figure 1) (3–5). The BFP is enclosed by a capsule and stabilized by fascial attachments, fibrous septa, and a rich vascular network, resulting in a multilocular and compartmentalized configuration (3, 4). Functionally, it serves as a gliding cushion during mastication and facial muscle movement, and contributes to facial contour and midfacial fullness (4, 5). Therefore, lesions involving the BFP may present with facial asymmetry and contour deformity. This compartmentalized architecture of the BFP may influence both the spatial behavior and treatment response of venous malformations, because lesions may extend along separated fat lobules and deep extensions rather than within a homogeneous tissue plane (3–5).

In a retrospective study of 109 primary pathologies involving the BFP, Li et al. identified vascular malformations as the largest disease category, accounting for 38 cases (34.9%), of which 37 were venous malformations (6). However, the literature on BFP venous malformations has consisted mainly of isolated case reports and small surgical series (7–16). Many lesions were initially reported as benign tumors, hemangiomas, arteriovenous malformations, or salivary gland-related lesions, and diagnosis was often established only after surgical excision (7–16). Accordingly, previous reports have focused primarily on pathological findings and surgical management, whereas the spatial extension patterns, compartmentalized behavior within the BFP, and implications for treatment planning have not been systematically evaluated.

Therefore, we retrospectively reviewed a cohort of patients with BFP venous malformations at our institution and performed a literature review to systematically characterize the anatomic extension patterns of these lesions. Based on the observed spatial behaviors, we proposed a classification framework and summarized the corresponding staged management considerations, including sclerotherapy, soft-tissue contouring, and skeletal reconstruction.

## Materials and methods

### Study design and patient selection

This retrospective study included consecutive patients with venous malformations (VMs) involving the buccal fat pad (BFP) as a major component who were treated at the Department of Oral and Maxillofacial Surgery, Peking University School and Hospital of Stomatology between January 2015 and December 2025. This study was approved by the Ethics Committee of Peking University School and Hospital of Stomatology (Approval No. PKUSSIRB-2026122077) and the requirement for informed consent was waived because anonymized retrospective data were used.

Inclusion criteria were as follows: (1) diagnosis of VM according to the ISSVA classification; (2) imaging-confirmed involvement of the BFP region on magnetic resonance imaging (MRI) and/or computed tomography (CT); (3) availability of complete imaging and follow-up data; and (4) completion of at least one treatment session with a minimum follow-up of 3 months. Fine needle aspiration cytopathology was performed when clinical and imaging findings were insufficient for diagnosis. Lesions confined to the subcutaneous tissue or parotid region without major BFP involvement were excluded.

### Anatomical assessment and classification

Axial and coronal fat-suppressed T2-weighted MRI images were used to evaluate lesion distribution and deep space involvement. CT images were reviewed when available to assess osseous remodeling and zygomatic asymmetry.

**Figure 2:**
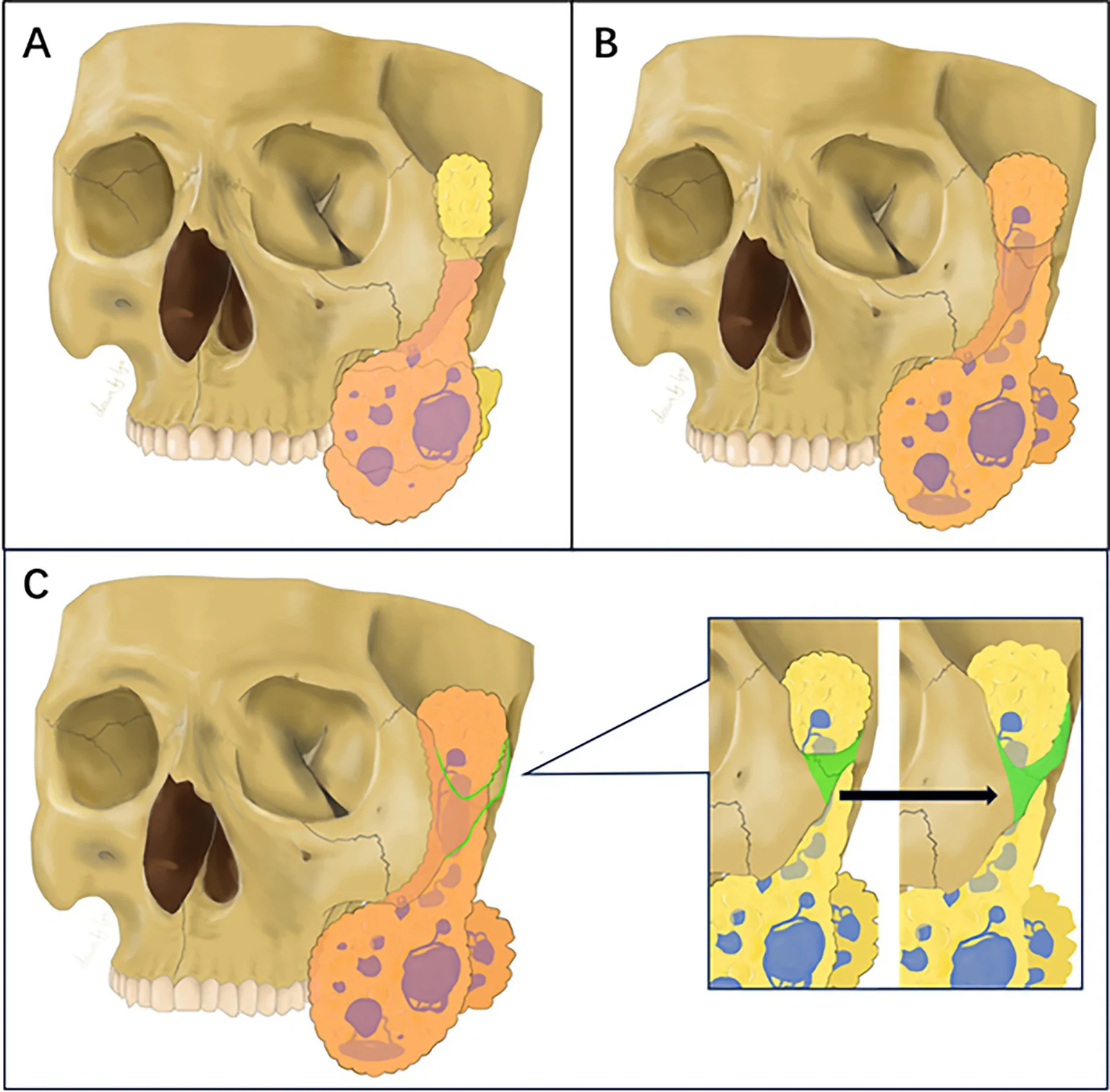
Extension-based classification of venous malformations involving the buccal fat pad. **(A)** Type I lesion confined predominantly to the body and buccal extension of the buccal fat pad. **(B)** Type II lesion extending beyond the buccal component into the temporal and pterygoid extensions and adjacent deep facial spaces, without clinically significant skeletal asymmetry. **(C)** Type III lesion with deep extension similar to Type II lesions and associated clinically significant zygomatic widening. The inset shows lateral displacement of the zygomatic contour.

**Figure 3:**
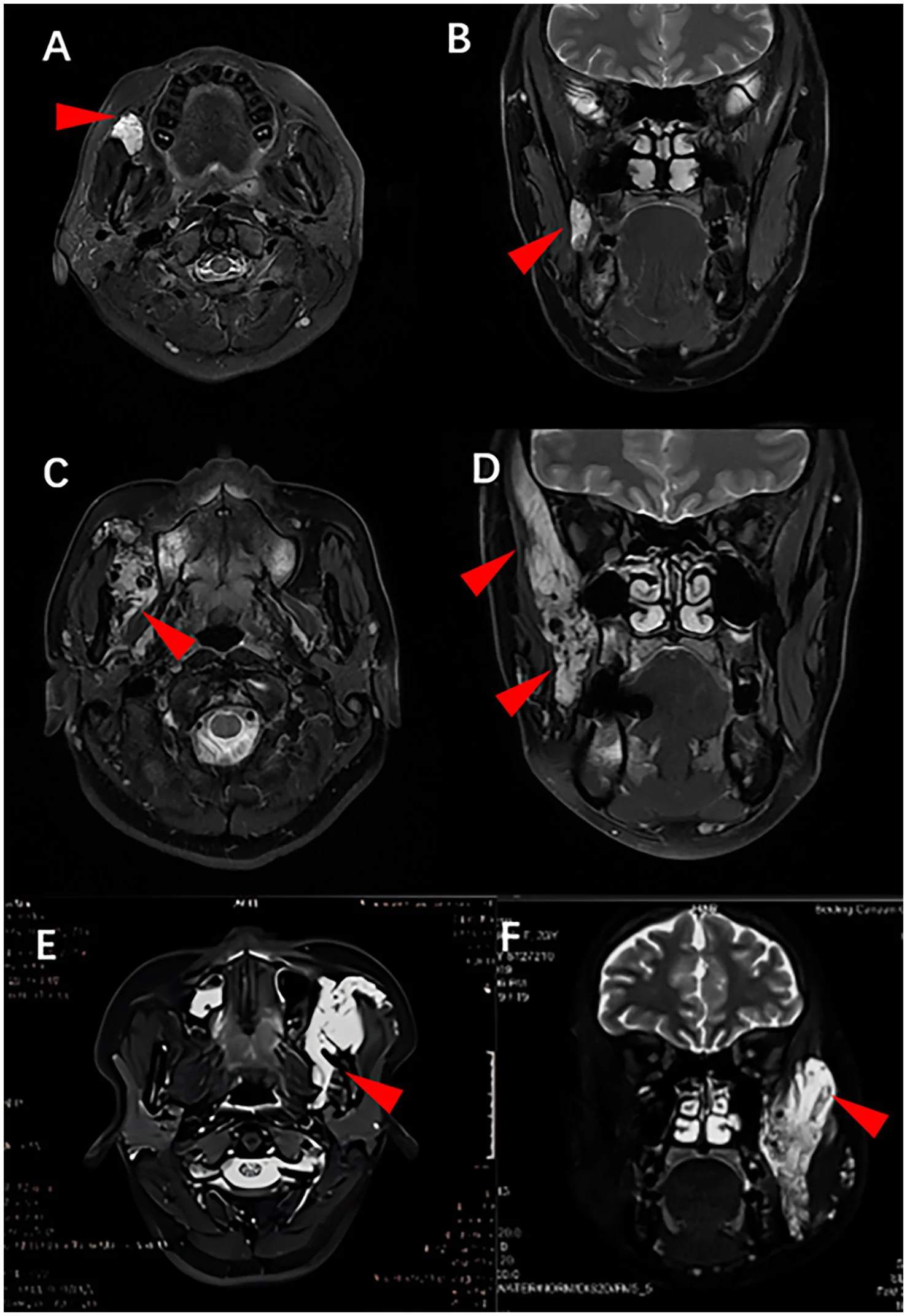
Representative MRI findings of the extension-based classification of buccal fat pad venous malformations. **(A,B)** Type I lesion confined predominantly to the body and buccal component of the buccal fat pad. **(C,D)** Type II lesion extending beyond the buccal component into adjacent deep facial spaces. **(E,F)** Type III lesion with extensive deep-space involvement and clinically significant zygomatic asymmetry.

Lesions were classified into three types according to reproducible imaging features and associated skeletal changes. Type I lesions were predominantly confined to the body of the BFP without involvement of the deep extensions and were usually below the lower rim of zygoma (Figures 2A, 3A,B). Type II lesions extended beyond the buccal body and superficial buccal extension to involve the deep extensions, including the temporal and pterygoid extensions, and typically extended into adjacent spaces such as the temporal, infratemporal, and pterygomandibular spaces (Figures 2B, 3C,D). Type III lesions were defined by clinically significant skeletal asymmetry or zygomatic widening on CT-based morphometric analysis (Figures 2C, 3E,F).

**Figure 4:**
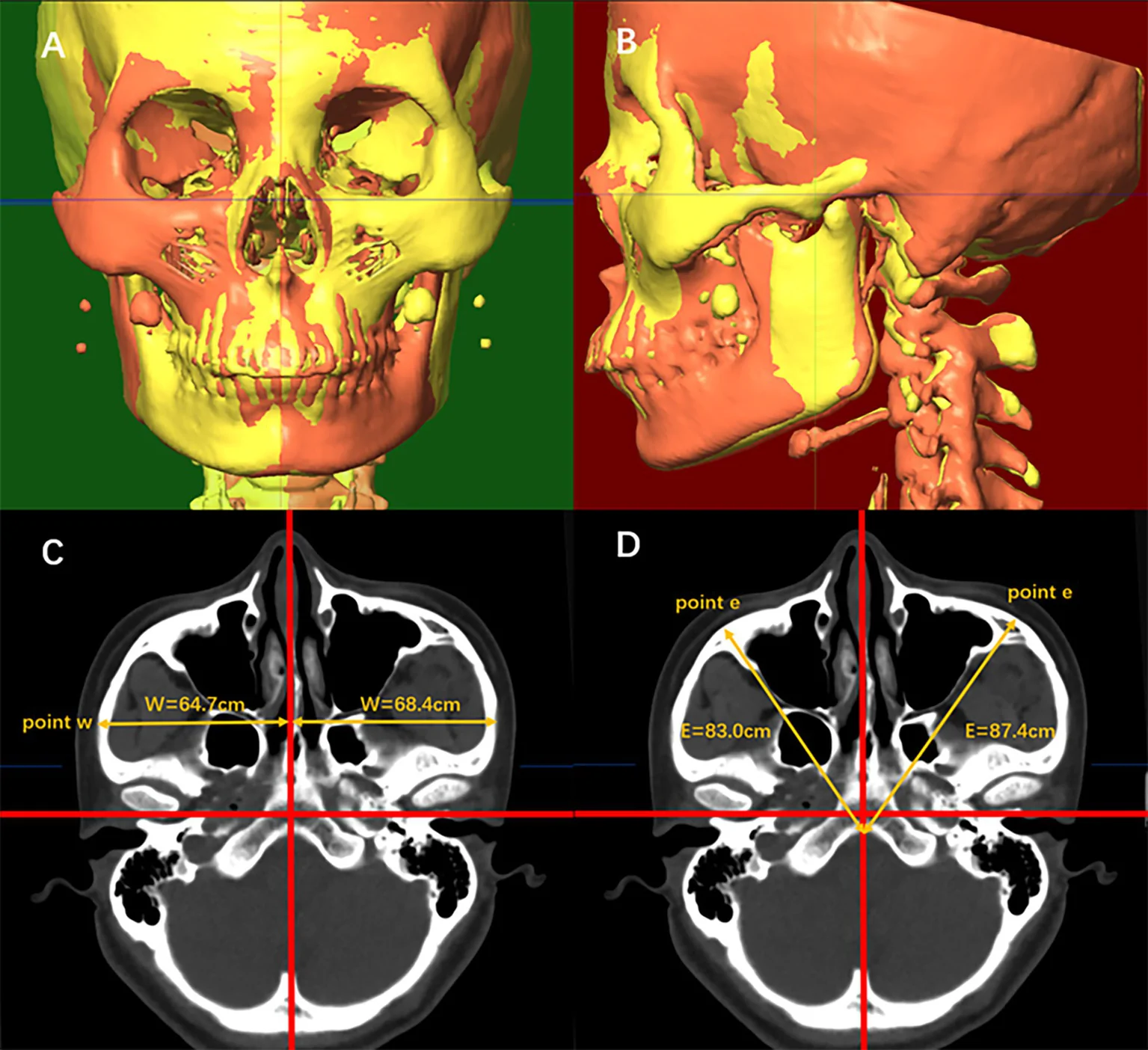
CT-based morphometric assessment of zygomatic asymmetry in a representative Type III lesion. **(A,B)** Three-dimensional CT reconstructions show mirrored craniofacial models and the midsagittal reference plane. **(C)** Zygomatic width (*W*) was measured from the midsagittal plane, and the bilateral width difference was recorded as *DW*. **(D)** Zygomatic eminence (*E*) was measured relative to the coronal reference plane, and the bilateral eminence difference was recorded as *DE*. *DE* exceeded the 4-mm threshold for clinically significant asymmetry.

For CT-based morphometric analysis, zygomatic width (W) and zygomatic eminence (E) were measured with reference to the midsagittal plane, using a method adapted from Gong et al. (Figure 4) (17). The absolute bilateral differences in width (DW) and eminence (DE) were calculated. A discrepancy of ≥4 mm in either DW or DE was considered to represent clinically significant zygomatic asymmetry. This threshold was selected as a pragmatic morphometric criterion because objective analyses of zygomaticomaxillary complex fractures have suggested that lateral zygomatic displacement exceeding approximately 4–5 mm is generally associated with clinically relevant facial asymmetry and commonly considered an indication for corrective surgery (18). Patients meeting this criterion were classified as Type III. Patients with visible but less pronounced asymmetry (<4 mm) were recorded as having mild asymmetry but were not classified as Type III.

All imaging studies were independently reviewed by one oral and maxillofacial radiologist and one oral and maxillofacial surgeon who had not participated in the treatment process.

### Treatment strategy and outcome assessment

**Figure 5:**
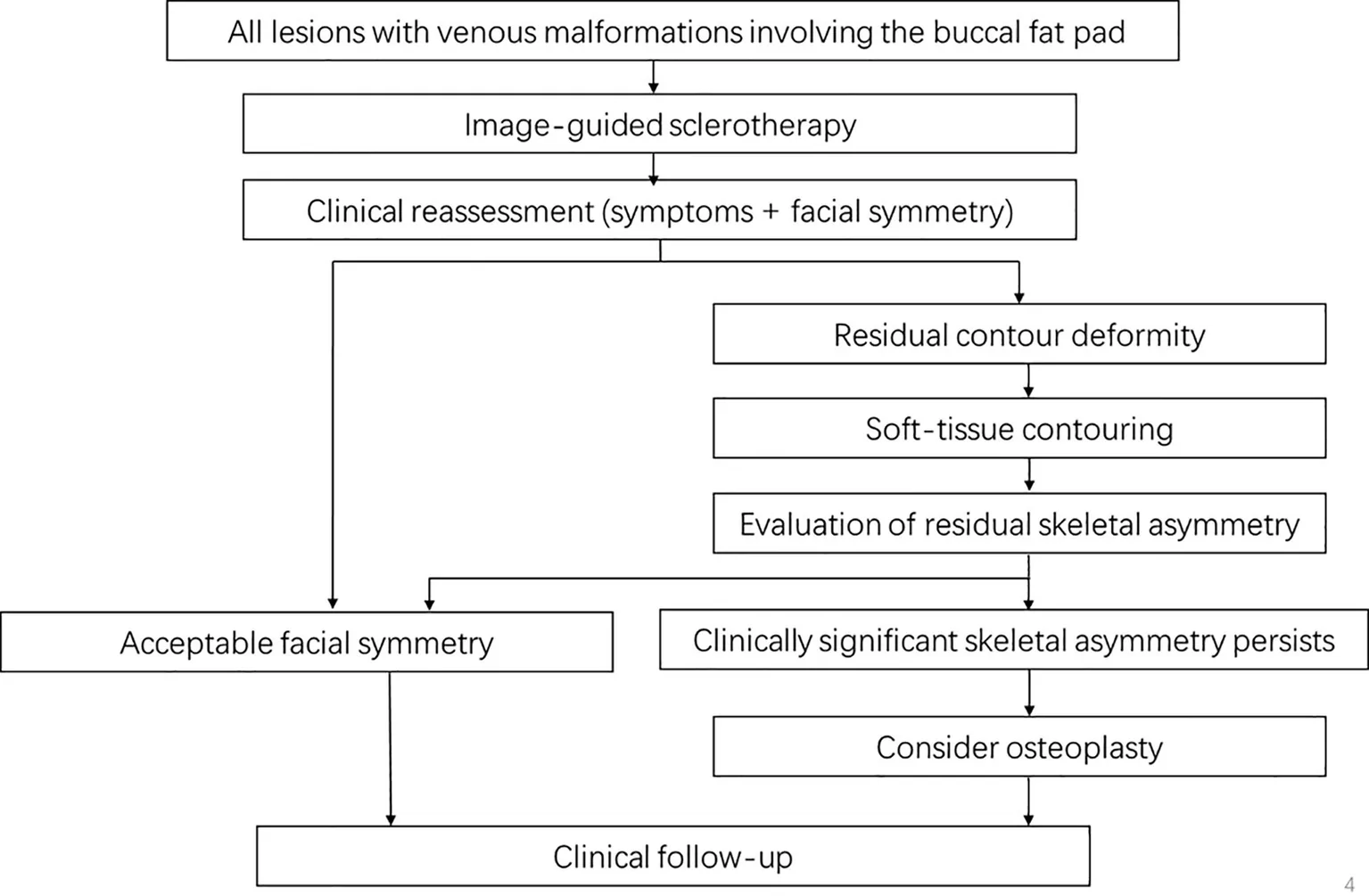
Staged treatment algorithm for venous malformations involving the buccal fat pad.

**Figure 6:**
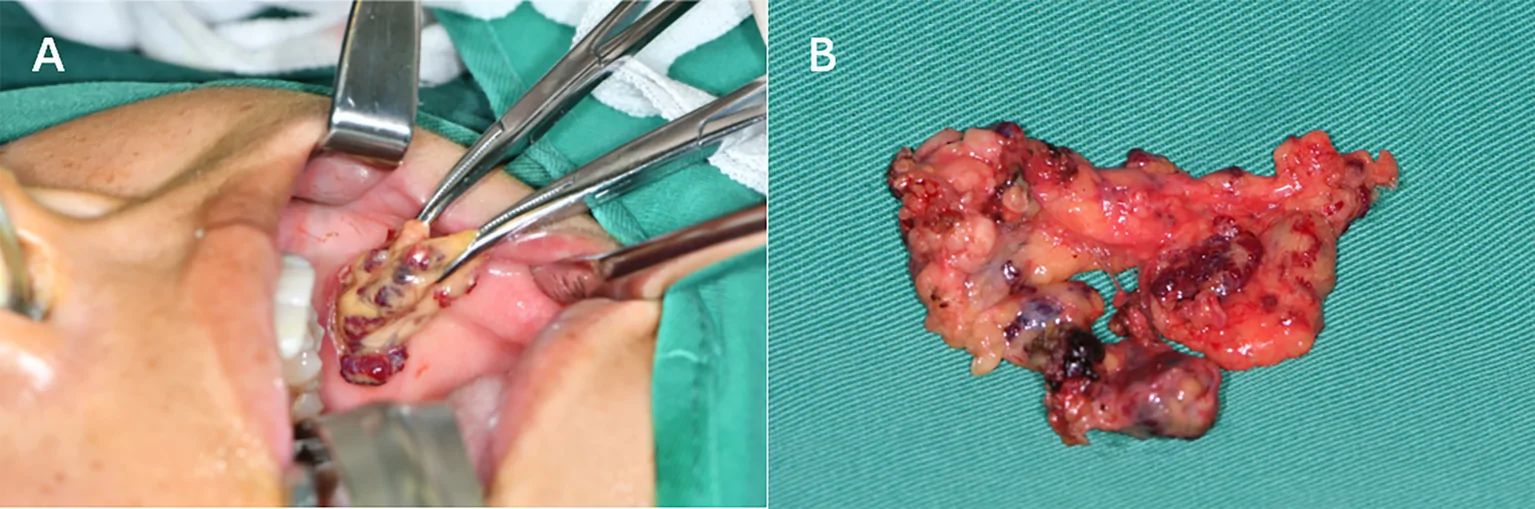
Intraoral soft-tissue contouring for residual facial asymmetry. **(A)** Intraoperative photograph shows partial resection of the residual venous malformation through an intraoral approach. **(B)** Photograph shows the resected fibrofatty and vascular tissue.

**Figure 7:**
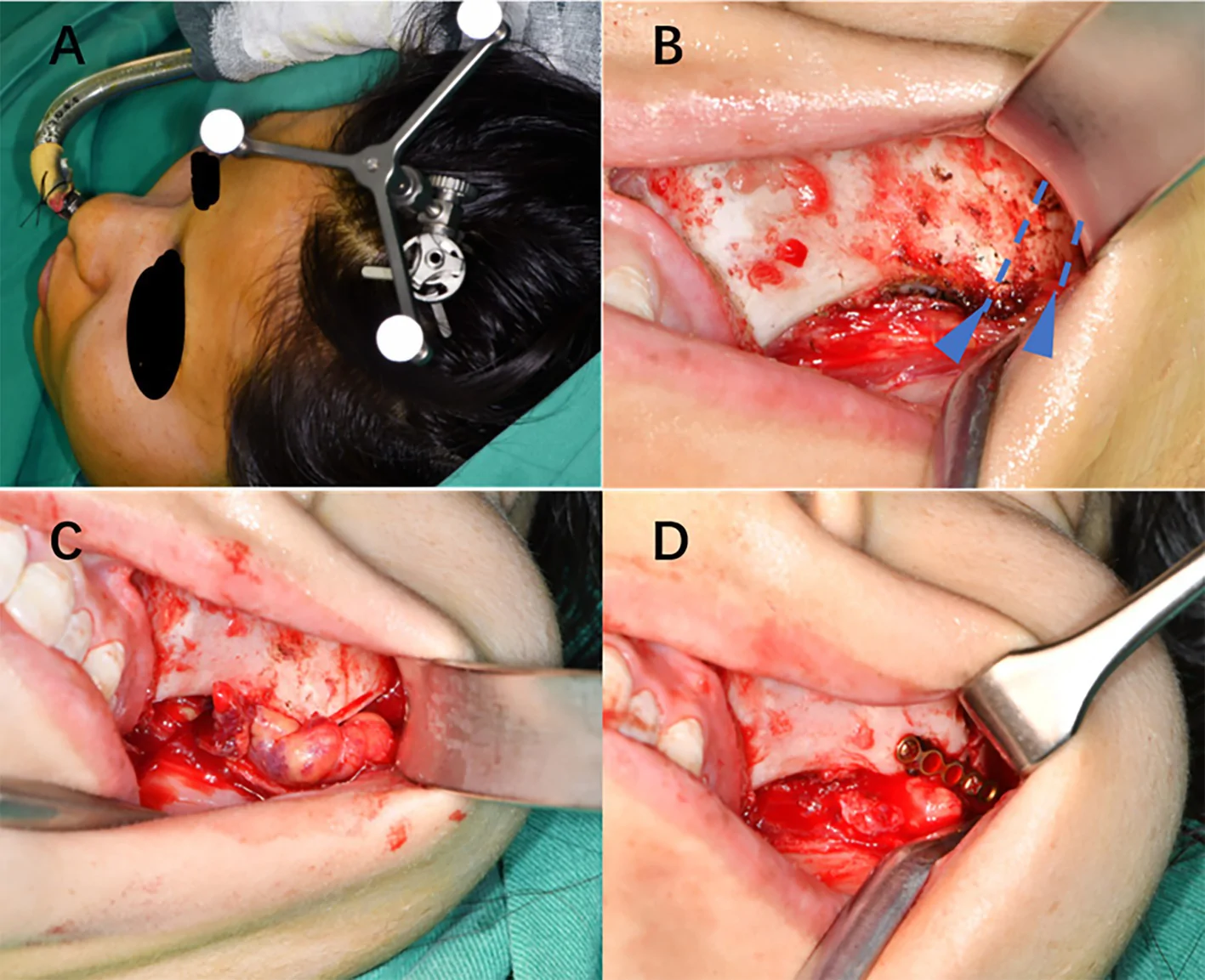
Navigation-assisted contouring osteoplasty for clinically significant zygomatic asymmetry. **(A)** Intraoperative photograph shows navigation setup based on preoperative virtual surgical planning. **(B)** Navigation image shows localization of the planned osteotomy line on the zygomatic arch. **(C)** Intraoperative photograph shows exposure of the zygomatic body and arch through an intraoral maxillary vestibular approach and resection of residual lesion on the medial aspect of the zygomatic arch. **(D)** Intraoperative photograph shows fixation of the repositioned zygomatic arch according to the preoperative digital plan.

All patients were managed according to a staged treatment strategy (Figure 5). Ultrasound-guided sclerotherapy was performed as the first-line treatment to control the vascular component of the lesion. Bleomycin or polidocanol foam was selected according to lesion characteristics and venous hemodynamic features. Polidocanol foam was preferentially used for lesions demonstrating positive postural enlargement tests and relatively rapid venous drainage, whereas bleomycin was generally selected for more localized lesions with negative postural tests and limited venous outflow. Treatment sessions were continued until no further technically feasible target venous spaces could be identified for effective ultrasound-guided puncture and sclerosant injection, indicating adequate control of the treatable vascular component. After stabilization of the vascular component, patients were reassessed for residual symptoms and facial symmetry. Selected patients with persistent soft-tissue asymmetry underwent partial lesion resection and soft-tissue contouring via an intraoral approach (Figure 6). Contouring osteoplasty was considered in patients with persistent clinically significant zygomatic asymmetry (Figure 7).

Treatment outcomes were assessed in terms of lesion-related symptoms and facial symmetry. Symptomatic outcomes were categorized as complete resolution or improvement according to clinical follow-up records. Facial symmetry outcomes were categorized as complete correction or improvement by comparison of pre- and post-treatment clinical photographs, with reference to clinical and imaging findings when available. Facial symmetry outcomes were independently assessed by two oral and maxillofacial surgeons and disagreements were resolved by consensus. Treatment-related complications and evidence of recurrence or progression were also recorded.

### Statistical analysis

Statistical analyses were performed using SPSS Statistics for Windows, version 26.0 (IBM Corp., Armonk, NY, USA). Continuous variables are presented as median (range), and categorical variables as frequencies and percentages. Because of the limited sample size and sparse categorical data, Fisher’s exact test was used to assess the association between lesion type and treatment escalation beyond sclerotherapy. All statistical tests were two-sided, and a *p* value < 0.05 was considered statistically significant.

### Literature review and anatomical reinterpretation

A targeted narrative review was conducted using a PubMed based search and supplemented by backward citation searching. PubMed was searched from database inception through March 2026 using combinations of the terms “buccal” or “buccal fat pad” with “venous malformation,” “arteriovenous malformation,” “vascular malformation,” “vascular lesion,” “hemangioma,” and “phlebolith.” Reference lists of eligible articles and relevant reviews were screened for additional reports including articles not indexed in PubMed.

Titles and abstracts were screened, followed by review of the full text or other available patient-level information. Case reports and case series were included when the lesion involved the BFP and sufficient information was available to assess lesion extent and anatomical extension patterns. Reports were excluded when BFP involvement could not be established, patient-level information was insufficient, or the same case had been reported more than once. Given the inconsistent terminology used in earlier literature, reports describing the lesion as a hemangioma or arteriovenous malformation were also assessed when the available findings supported reinterpretation as a VM. Eligible cases were reinterpreted according to the extension-based framework used in the present study.

The review was used to determine whether the extension patterns observed in the present cohort were also recognizable in published cases and to summarize reported management strategies, rather than to provide formal external validation of the classification framework.

## Results

### Patient characteristics

A total of 22 patients with VMs involving the BFP were included. The cohort comprised 14 females and 8 males, with a median age of 20 years (range, 8–78 years). The median follow-up duration was 11.5 months (range, 3–77 months). Clinical and imaging characteristics are summarized in Table 1.

**Table 1:** Clinical and imaging characteristics of buccal fat pad venous malformations.

Case	Age/sex	Presenting symptoms	Buccal and body extensions	Pterygomandibular involvement	Infratemporal involvement	Associated vascular malformations	Skeletal asymmetry	Type
1	10/F	Pain/swelling	✓	–	–	None	None	Type I
2	78/F	Swelling	✓	–	–	None	None	Type I
3	40/M	Fullness with positional enlargement	✓	–	–	None	None	Type I
4	19/F	Swelling	✓	–	–	None	None	Type I
5	37/F	Palpable nodule	✓	–	–	None	None	Type I
6	48/F	Swelling	✓	–	–	None	None	Type I
7	8/F	Swelling with positional enlargement	✓	–	–	None	None	Type I
8	33/F	Swelling with positional enlargement	✓	✓	✓	Intradermal cheek venous malformation and intraparotid venous malformation on the ipsilateral side	Mild	Type II
9	30/M	Swelling	✓	–	✓	Intramuscular venous malformation of the contralateral masseter muscle	None	Type II
10	39/F	Swelling	✓	✓	✓	None	None	Type II
11	16/M	Swelling with positional enlargement	✓	✓	✓	Venous malformation of the bilateral parapharyngeal spaces	Mild	Type II
12	20/F	Swelling with positional enlargement	✓	✓	✓	None	Mild	Type II
13	17/F	Swelling	✓	–	✓	None	None	Type II
14	29/F	Swelling	✓	✓	✓	None	Mild	Type II
15	40/F	Swelling	✓	✓	–	None	None	Type II
16	15/M	Swelling	✓	✓	✓	None	Mild	Type II
17	18/F	Swelling	✓	✓	✓	None	None	Type II
18	45/M	Swelling with positional enlargement	✓	✓	✓	None	Clinically significant	Type III
19	16/M	Swelling with positional enlargement	✓	✓	✓	Venous malformations of the bilateral parapharyngeal spaces and soft palate	Clinically significant	Type III
20	26/F	Swelling with positional enlargement	✓	✓	✓	None	Clinically significant	Type III
21	15/M	Swelling with positional enlargement	✓	✓	✓	None	Clinically significant	Type III
22	20/M	Swelling with positional enlargement	✓	✓	✓	Lymphatic malformation of the tongue and cheek, with arteriovenous malformation of the pterygomandibular space	Clinically significant	Type III

Facial contour alteration was the predominant clinical manifestation. Twenty-one patients presented with cheek swelling or facial asymmetry, including nine who reported position-dependent enlargement. Pain was reported in one patient, and a palpable cheek nodule was identified in another. Five patients had multifocal craniofacial venous malformations with concurrent involvement of anatomical sites beyond the BFP, including the parapharyngeal space, tongue, masseter muscle, parotid gland, and soft palate.

### Anatomical extension patterns and classification

Imaging review showed that seven lesions (31.8%) were predominantly confined to the BFP body and buccal extension, whereas 15 lesions (68.2%) extended beyond the buccal compartment (Table 1). Among extension-type lesions, combined infratemporal and pterygomandibular extension was the predominant pattern (12 of 15 lesions).

Zygomatic asymmetry was observed in both Type II and Type III lesions. Mild asymmetry was observed in five patients with Type II lesions, whereas clinically significant zygomatic widening meeting the predefined morphometric criteria was identified in five patients who were classified as having Type III lesions.

According to the proposed framework, seven lesions were categorized as Type I, 10 as Type II, and five as Type III (Figure 3). The relationship between lesion type, treatment escalation, and facial symmetry outcomes is summarized in Table 2.

**Table 2:** Treatment escalation and facial symmetry outcomes across lesion types.

Type	*n*	Sclerotherapy alone (*n*)	Soft-tissue contouring (*n*)	Osteoplasty (*n*)	Treatment escalation (*n*)	Restored symmetry	Improved symmetry
I	7	7	0	0	0/7 (0%)	6 (85.7%)	1 (14.3%)
II	10	4	6	0	6/10 (60%)	8 (80.0%)	2 (20.0%)
III	5	0	5	3	5/5 (100%)	1 (20.0%)	4 (80.0%)

### Treatment strategies

All patients received image-guided sclerotherapy as the initial stage of treatment (Table 3). Bleomycin or polidocanol foam was used as the sclerosant. Overall, 77 sclerotherapy sessions were performed, with a median of 3.5 sessions per patient (range, 1–8). Some patients had received sclerotherapy before referral to our institution.

**Table 3:** Staged treatment strategies and outcomes of buccal fat pad venous malformations.

Case	Type	Previous treatment	No. of sclerotherapy sessions	Soft-tissue contouring	Osteoplasty	Symptom outcome	Facial symmetry outcome	Complications	Additional management
1	I	None	1	–	–	Resolved	Restored	None	None
2	I	None	1	–	–	Resolved	Restored	None	None
3	I	None	2	–	–	Resolved	Improved	None	None
4	I	None	2	–	–	Resolved	Restored	None	None
5	I	None	4	–	–	Resolved	Restored	None	None
6	I	None	1	–	–	Resolved	Restored	None	None
7	I	None	5	–	–	Improved	Restored	None	None
8	II	Sclerotherapy and temporal lesion resection	3	✓	–	Resolved	Restored	Temporary facial nerve weakness	None
9	II	None	3	–	–	Resolved	Improved	None	None
10	II	None	4	✓	–	Resolved	Restored	None	None
11	II	None	4	✓	–	Resolved	Restored	Hematoma	None
12	II	None	5	–	–	Resolved	Restored	None	None
13	II	None	3	✓	–	Resolved	Restored	None	None
14	II	None	4	–	–	Improved	Improved	None	None
15	II	None	5	–	–	Resolved	Restored	None	None
16	II	None	5	✓	–	Resolved	Restored	None	None
17	II	None	4	–	–	Resolved	Restored	None	None
18	III	Multiple sessions of sclerotherapy	3	✓	–	Resolved	Improved	None	Declined osteoplasty
19	III	None	3	✓	–	Improved	Improved	None	Ongoing treatment
20	III	Multiple sessions of sclerotherapy	2	✓	✓	Improved	Improved	None	None
21	III	None	3	✓	✓	Resolved	Restored	Wound infection	None
22	III	None	8	✓	✓	Improved	Improved	Progression of residual deep arteriovenous malformation	None

After stabilization of the vascular component, further treatment was determined according to residual contour deformity. Ten patients (45.5%) underwent soft-tissue contouring through an intraoral approach to improve persistent facial asymmetry, including five with Type II lesions and five with Type III lesions. No patient with a Type I lesion required contouring surgery.

Among the five Type III lesions, three underwent additional osteoplasty for correction of clinically significant zygomatic widening. One patient declined further skeletal correction after achieving satisfactory symptomatic improvement, and one remained under active treatment at the last follow-up. No patient with Type I or Type II lesions underwent osteoplasty. Treatment escalation according to lesion type is summarized in Table 2.

### Clinical outcomes and complications

Clinical outcomes are summarized in Table 3. Lesion-related symptoms completely resolved in 17 patients (77.3%) and improved in the remaining five (22.7%). Complete correction of facial asymmetry was achieved in 15 patients (68.2%), whereas seven patients demonstrated improvement with residual asymmetry. Post-treatment MRI demonstrated reduction of the vascular component in all patients, although quantitative volumetric analysis was not performed.

**Figure 8:**
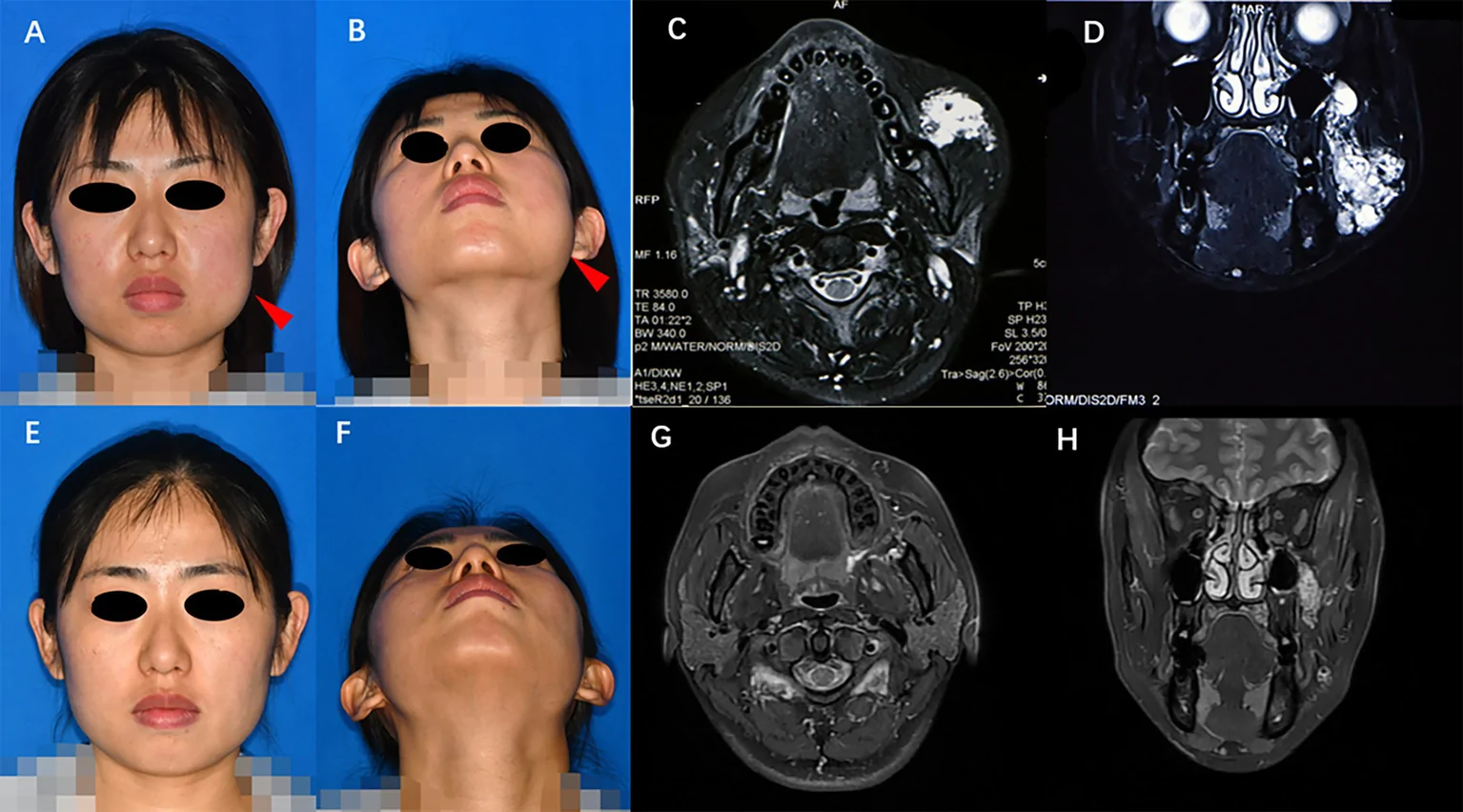
Representative type II lesion before and after treatment. **(A,B)** Pre-treatment clinical photographs show cheek fullness and facial asymmetry. **(C,D)** Pre-treatment MR images show extension of the lesion into adjacent deep facial spaces. **(E,F)** Post-treatment clinical photographs show improved facial symmetry. **(G,H)** Post-treatment MR images show decreased lesion extent.

Among the seven patients with Type I lesions, six achieved complete correction after sclerotherapy alone and one showed partial improvement. Among the 10 patients with Type II lesions, seven achieved complete correction, including four who underwent subsequent soft-tissue contouring, whereas the remaining three showed partial improvement. A representative Type II lesion demonstrating reduction of lesion extent and improvement of facial symmetry after staged treatment is shown in Figure 8.

Among the five patients with Type III lesions, three underwent additional osteoplasty for correction of clinically significant zygomatic widening. One patient declined further skeletal correction after achieving satisfactory symptomatic improvement, and one remained under active treatment at the last follow-up. No patient with Type I or Type II lesions underwent osteoplasty. The requirement for treatment escalation beyond sclerotherapy increased progressively across lesion types (Type I: 0/7, Type II: 6/10, and Type III: 5/5; Fisher’s exact test, *p* = 0.0017) (Table 2).

**Figure 9:**
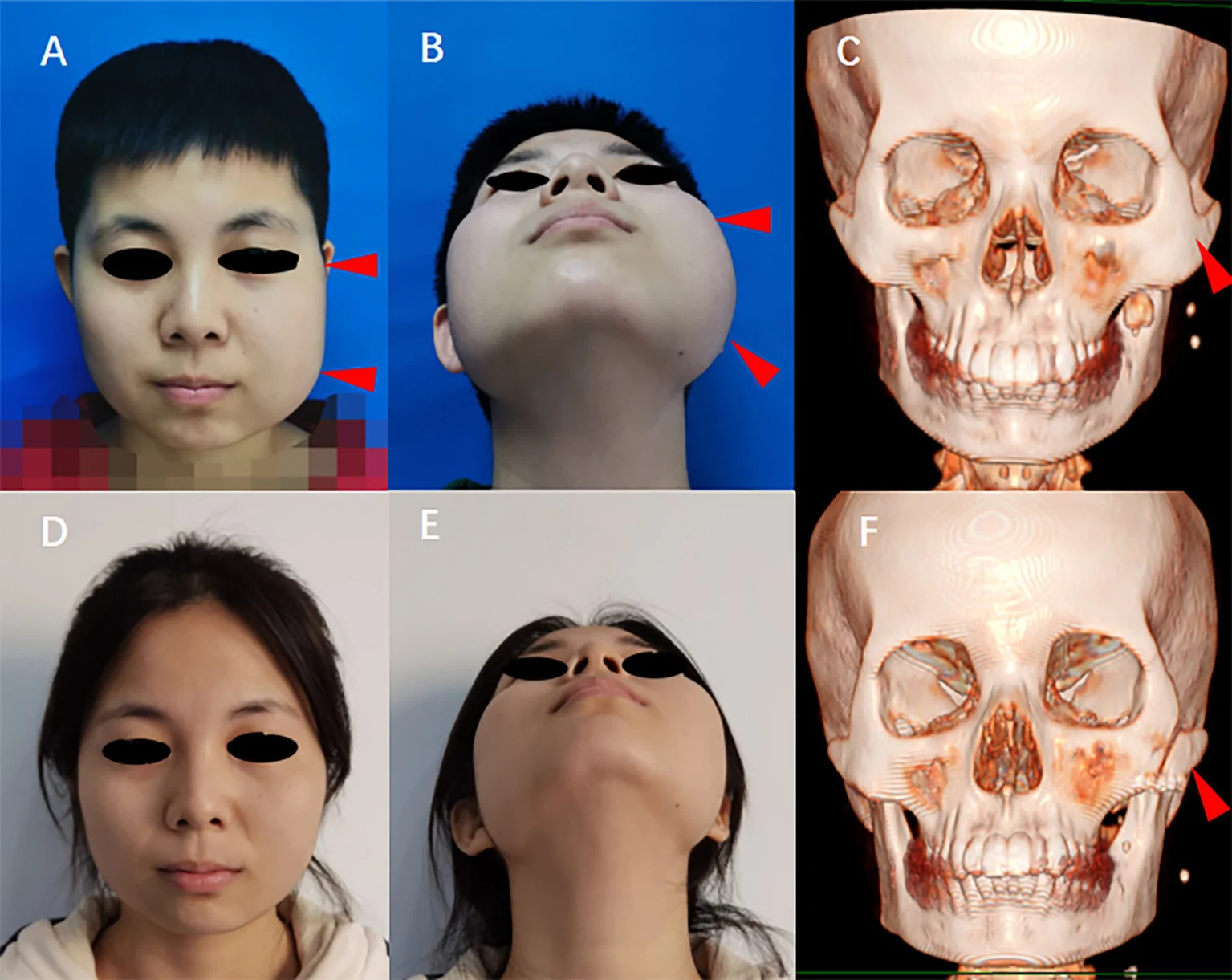
Representative Type III lesion before and after treatment. **(A,B)** Pre-treatment clinical photographs show facial asymmetry. **(C)** Pre-treatment three-dimensional CT reconstruction shows zygomatic widening on the affected side. **(D,E)** Post-treatment clinical photographs show improved facial symmetry. **(F)** Post-treatment three-dimensional CT reconstruction shows correction of zygomatic asymmetry after contouring osteoplasty.

No major complications related to sclerotherapy were observed. Three surgical complications occurred after partial resection of the buccal fat pad, including temporary facial nerve weakness in one patient, postoperative hematoma in one patient, and postoperative wound infection in one patient. One patient experienced progression of a deep vascular lesion during follow-up. Further evaluation suggested that the progression was associated with a previously unrecognized deep arteriovenous malformation component rather than recurrence or progression of the treated buccal fat pad venous malformation (Figure 9).

### Literature review and anatomical reinterpretation

A total of 12 previously reported cases of VMs involving the BFP were identified and reinterpreted according to the extension-based framework used in the present study (Table 4). Five lesions were categorized as Type I, six as Type II, and one as Type III.

**Table 4:** Anatomical reinterpretation of previously reported buccal fat pad venous malformations.

Reference	Patient	Buccal involvement	Temporal extension	Deep extension	Skeletal asymmetry	Reinterpreted type	Non-surgical treatment
Ikegami and Nishijima (1984) (7)	23/M	✓	–	–	None	Type I	None
Sano K et al. (1988) (8)	37/F	✓	–	–	None	Type I	None
Sano K et al. (1988) (8)	17/M	✓	✓	✓	Present	Type III	None
Ono M et al. (1994) (9)	22/F	✓	–	✓	None	Type II	None
Hassani A et al. (2014) (10)	28/F	✓	–	✓	None	Type II	Triamcinolone injection
Saha A et al. (2015) (11)	12/M	✓	–	–	None	Type I	None
Watanabe S et al. (2017) (12)	54/F	✓	–	✓	None	Type II	None
Yamamoto H et al. (2017) (13)	28/F	✓	–	✓	None	Type II	None
Baldin AV et al. (2018) (14)	31/F	✓	–	–	None	Type I	Sclerotherapy
Baldin AV et al. (2018) (14)	65/F	✓	✓	✓	Present (mild)	Type II	None
Berenguer B et al. (2019) (15)	12/F	✓	–	–	None	Type I	Percutaneous cryotherapy
Kato H et al. (2023) (16)	47/F	✓	–	–	None	Type I	None

Among the seven non-Type I lesions, deep anatomical extension was consistently identified, involving the infratemporal, pterygomandibular and temporal regions. Temporal extension was reported in two cases, both accompanied by additional deep-space involvement. One of these patients also demonstrated facial skeletal asymmetry and was categorized as Type III. Overall, the extension patterns identified in published cases resembled those observed in the present cohort, particularly the tendency for lesions to extend beyond the buccal component into adjacent deep facial spaces.

Management strategies reported in the literature differed from the strategy used in the current series. Surgical excision was the predominant treatment modality, and an intraoral approach was used in all but one surgically treated case. Non-surgical treatment was reported in only three cases, including sclerotherapy, triamcinolone injection, and percutaneous cryotherapy. An extraoral approach was used only in the patient categorized as Type III.

Taken together, these findings suggest that extension beyond the buccal component of the BFP was recognizable in most previously reported cases, and the anatomical patterns in published cases were broadly consistent with the present cohort.

## Discussion

To the best of our knowledge, this study represents the largest clinicoradiological series of VMs involving the buccal fat pad. Three principal findings emerged. First, BFP VMs demonstrated reproducible patterns of anatomical extension, most commonly toward the infratemporal and pterygomandibular regions. Second, increasing anatomical extent was associated with progressively more complex treatment strategies, ranging from sclerotherapy alone to adjunctive soft-tissue contouring and, in selected patients, skeletal correction. Third, similar extension patterns could be retrospectively identified in previously reported cases, suggesting that these spatial behaviors may represent characteristic anatomical features of BFP VMs rather than isolated observations (3, 4, 7–16, 19).

The observed extension pattern is anatomically plausible. The predominance of infratemporal and pterygomandibular involvement in the present cohort corresponds closely to the anatomical organization of the BFP. Although the buccal extension contributes substantially to cheek fullness, the deeper extensions of the BFP communicate directly with adjacent deep facial spaces, including the infratemporal and pterygomandibular regions (3, 19). Within this multilobular and compartmentalized architecture, VMs may extend along interconnected adipose lobules and deep extensions that are separated by fascial attachments and fibrous septa. This compartmentalized pattern of growth may explain not only the frequent deep-space involvement observed in the present cohort but also the progressive contour deformity and skeletal asymmetry seen in more extensive lesions (3, 4, 19).

On this basis, the proposed extension-based classification should be regarded as a descriptive anatomical framework rather than as a rigid staging system or treatment algorithm. Derived from clinicoradiological observations in a single-center cohort, it characterizes progressively increasing anatomical extension, from lesions confined to the buccal component of the BFP to those involving adjacent deep spaces and, ultimately, clinically significant skeletal asymmetry. Rather than prescribing specific interventions, this framework is intended to facilitate radiological reporting, multidisciplinary communication, and patient counseling regarding the anticipated complexity of staged management.

Exploratory analysis demonstrated a significant association between lesion type and treatment escalation, supporting the relationship between anatomical extent and management complexity. This association should not be interpreted as evidence that the classification predicts specific interventions. Although osteoplasty was performed more frequently in Type III lesions, this reflected the presence of clinically significant skeletal deformity within this phenotype. Decisions regarding soft-tissue contouring or osteoplasty were individualized after adequate control of the vascular component.

The three categories should therefore be viewed as points along a clinicoradiological continuum rather than as biologically discrete entities. Existing VM classifications primarily describe flow characteristics and pathological type and they provide limited information on how a lesion spreads within specific anatomical spaces. The present framework complements current systems by emphasizing the anatomical extension pattern of BFP VMs, thereby providing additional information for treatment planning (20).

The management implications of this framework are consistent with contemporary principles for head and neck VMs. Sclerotherapy is widely regarded as a major first-line treatment for most low-flow venous and lymphatic malformations of the head and neck, although the optimal sclerosant and treatment schedule remain lesion- and center-dependent (1, 21, 22). In the present cohort, image-guided sclerotherapy was used as the initial treatment in all patients to control the dominant venous component, reduce lesion volume, and improve lesion-related symptoms. This approach was sufficient in most Type I lesions, in which the vascular component was largely limited to the buccal compartment. In Type II and Type III lesions, however, stabilization of the vascular component did not always translate into complete restoration of facial symmetry. For these patients, partial lesion removal provided a reasonable option to correct persistent soft-tissue asymmetry while avoiding radical extirpation of the entire lesion, which would require extensive removal of BFP, and potentially increase complication risks. In Type III lesions, zygomatic widening represented a skeletal contributor to facial asymmetry that could not be corrected by sclerotherapy or soft-tissue contouring alone; therefore, contouring osteoplasty was considered selectively when residual skeletal asymmetry remained clinically significant and the patient desired further correction. Thus, the treatment pathway observed in this cohort should be interpreted as a consequence of progressively greater anatomical involvement and its associated contour deformities, rather than as a treatment strategy dictated by the classification itself (1, 21–23).

The contrast between the present management strategy and the published literature is also informative. Most previously reported BFP vascular lesions were managed surgically, usually through an intraoral approach, and many were diagnosed definitively only after excision and histopathological examination (7–16). Several factors may explain this pattern. First, many historical reports used the term “hemangioma” before widespread acceptance of the ISSVA classification, and some lesions would likely be reclassified as venous or other vascular malformations under current terminology (7–16, 20). Second, BFP vascular lesions may mimic other cheek masses, salivary lesions, or calcified soft-tissue lesions. For example, phlebolith-containing vascular lesions may clinically resemble sialolithiasis, whereas deep lesions extending toward the pterygopalatine fossa may be mistaken for neurogenic or salivary gland tumors (11, 12, 24). Third, the deep and compartmentalized location of the BFP may limit the reliability of aspiration and complicate preoperative diagnosis in selected cases. Consequently, the high proportion of surgical treatment in earlier reports should not be interpreted as evidence that primary excision is the preferred treatment for all BFP VMs. Rather, it more likely reflects diagnostic uncertainty, publication bias toward unusual surgically treated cases, and the historical evolution of vascular anomaly classification and imaging-based management (7–16, 20, 24).

Symptom control and restoration of facial symmetry did not always occur in parallel. Lesion-related symptoms generally resolved or improved, whereas complete correction of facial asymmetry became progressively more difficult with increasing anatomical extent. This distinction has therapeutic implications. Sclerotherapy remains the primary treatment for BFP VMs and is directed toward maximal control of the treatable vascular component. Because of the compartmentalized anatomy of the buccal fat pad, residual imaging abnormalities after treatment may represent fibrosis, fibro-fatty remodeling, skeletal remodeling, or small residual venous spaces that are no longer amenable to effective image-guided sclerotherapy. Adjunctive soft-tissue contouring or osteoplasty was subsequently performed in selected patients to address persistent contour deformity after satisfactory vascular control. Treatment success should therefore be defined by durable symptom control together with restoration of a facial contour acceptable to the patient, rather than complete radiographic normalization. During the observed follow-up period, residual imaging abnormalities did not require additional intervention because of progression of the treated BFP lesion. One patient underwent further treatment because of a previously unrecognized associated deep arteriovenous malformation rather than recurrence of the treated BFP VM. The long-term behavior of residual vascular components remains uncertain. These findings support an individualized management strategy that balances anatomical extent, residual deformity, treatment morbidity, and patient expectations (1, 21–23).

This study has several limitations. It was retrospective, single-center, and included a relatively small cohort with limited follow-up. Treatment outcomes were assessed qualitatively, and quantitative facial symmetry analysis, patient-reported outcome measures, and MRI volumetric assessment were not performed, although all patients demonstrated radiological reduction of the vascular component on follow-up MRI. Reinterpretation of previously published cases was constrained by the quality of the available clinical and imaging documentation. Nevertheless, comparable extension patterns were consistently identified in both the present cohort and published cases. Further multicenter studies incorporating standardized imaging, quantitative outcome assessment, and longer follow-up are warranted to determine its reproducibility, clinical applicability, and long-term utility.

## Conclusion

Venous malformations involving the buccal fat pad show characteristic extension patterns rather than random expansion. Understanding these anatomical behaviors may help guide staged treatment and improve management of this uncommon entity.

## Data Availability

The datasets presented in this article are not readily available because the raw clinical and imaging data are not publicly available because they contain potentially identifiable patient information. Access is restricted due to patient privacy and ethical restrictions. Requests to access the datasets should be directed to Yunan Liu, liuyunan860326@126.com.
